# 

**Published:** 1806-07

**Authors:** 


### MEDICAL AND PHYSICAL

### JOURNAL.

### THE MEDICAL AND PHYSICAL JOURNAL.

CONDUCTED BY

T. BRADLEY, M. D.

AND

R. BATTY, M. D.

VOL. XVI.

FROM JUNE TO DECEMBER, 1806.

LIBRARY
Washington, D.C.
5122

*LONDON:*

PRINTED FOR RICHARD PHILLIPS, NO. 6, BRIDGE-STREET, BLACKFRIARS.

By William Thorne, Red Lion Court, Fleet Street.

